# The Ethics of Clinical Research on Diseases of the Fetus and Newborn: Balancing Benefit–Risk, Autonomy, and Maternal–Fetal Interests

**DOI:** 10.1055/a-2764-2202

**Published:** 2025-12-30

**Authors:** Robert M. Nelson, Karla Childers, Leona E. Ling, Lori Alquier, Yosuke Komatsu, Lisa Schwartz, Anne M. Stevens, May Lee Tjoa, Kenneth J. Moise, Sara F. Goldkind

**Affiliations:** 1 Pediatric Drug Development, Child Health Innovation Leadership Department, Johnson & Johnson, Raritan, New Jersey, United States; 2 Bioethics-Based Science and Technology Policy, Office of the Chief Medical Officer, Johnson & Johnson, New Brunswick, New Jersey, United States; 3 Translational Science (Autoantibody), Johnson & Johnson, Cambridge, Massachusetts, United States; 4 Global Regulatory Affairs, Johnson & Johnson, Raritan, New Jersey, United States; 5 Department of Maternal Fetal Immunology, Johnson & Johnson, Spring House, Pennsylvania, United States; 6 Department of Immunology, Johnson & Johnson, Raritan, New Jersey, United States; 7 Department of Clinical Development, Century Therapeutics, Seattle, Washington, United States; 8 Global Medical Affairs, Johnson & Johnson, Cambridge, Massachusetts, United States; 9 Department of Women's Health, Dell Medical School, Austin, Texas, United States; 10 Goldkind Consulting, LLC, Chevy Chase, Maryland, United States

**Keywords:** pregnancy, fetal and neonatal alloimmune thrombocytopenia, hemolytic disease of the fetus and newborn, ethics, research, nipocalimab, Fc receptor, neonatal

## Abstract

**Objective:**

Pregnant persons historically have been excluded from clinical trials. Recently, there has been a shift from exclusion toward inclusion of pregnant persons in research while acknowledging the complexity of reproductive ethics and the intertwined interests of the pregnant person and fetus. Our objective was to use a principle-based approach to review the ethics of clinical research concerning pregnancy-related disorders that predominantly affect fetal well-being.

**Study Design:**

Ethical principles are applied to the design of interventional trials in two rare conditions of pregnancy, hemolytic disease of the fetus and newborn (HDFN) and fetal and neonatal alloimmune thrombocytopenia (FNAIT).

**Results:**

Severe HDFN and FNAIT are fetal and neonatal diseases caused by maternal alloantibodies with potential outcomes including maternal and neonatal morbidity, preterm delivery, and fetal or neonatal death. Early-onset severe HDFN was the initial indication for a phase 2 open-label study of nipocalimab in pregnancy, given poor outcomes after prior severe HDFN pregnancy(s). After establishing proof of concept, a phase 3 randomized placebo-controlled study was initiated based on the ethical principles of equipoise and generating socially valuable data to support the efficacy and safety of nipocalimab in severe HDFN. However, off-label use of antenatal intravenous immunoglobulin (IVIg) in standard-risk FNAIT pregnancies made a randomized placebo-controlled study challenging. Thus, the FNAIT clinical program for nipocalimab in pregnant persons with standard-risk FNAIT includes a randomized, placebo-controlled study limited to sites that do not use antenatal IVIg, and a global randomized open-label study of nipocalimab or IVIg.

**Conclusion:**

Knowledge of the clinical course and management of severe HDFN and FNAIT, the non-clinical and non-pregnant human clinical evidence, and input from physicians, patients, and health authorities permitted the design of clinical protocols that satisfy the principles of beneficence and non-maleficence in these rare and complex diseases.

**Key Points:**

- Ethical principles apply to pregnancy-related disorders affecting fetal well-being.
- Placebo-controlled randomized trials in HDFN are based on equipoise.
- FNAIT programs must account for the off-label use of IVIg.

## Introduction

Pregnant persons have historically been excluded from clinical trials for conditions unrelated to pregnancy, partly due to concerns about risks to the fetus. 1 While this approach aimed at protecting pregnant persons and their fetuses, they and their health care providers are left with inadequate data to provide guidance on medication use during pregnancy. 2 Consequently, pregnant persons lack access to otherwise safe and effective medications for treating their chronic disease during pregnancy. 3 4 Pregnant persons do participate in research on pregnancy-related disorders (e.g., preeclampsia, labor induction), 5 6 including hemolytic disease of the fetus and newborn (HDFN) and fetal and neonatal alloimmune thrombocytopenia (FNAIT). This ongoing research, along with the recent shift toward including pregnant persons more broadly to inform medical decision-making, highlights the need to balance safety with advancing medical knowledge to ensure healthy pregnancies. 7 8

Historically, reproductive ethics has been framed as “maternal–fetal conflict.” 9 The “fetus as patient” terminology elevated the fetus to a position of equal or superior status to the pregnant person, 10 raising complex ethical questions about the balance of interests and rights between the pregnant person and the fetus. Often, pregnant persons have been regarded as “vectors of disease,” 11 such as HIV, or as a passive “environment” for the fetus, 12 rather than as autonomous persons with their own health and rights. These approaches overlook the complexity of reproductive ethics and the need for a more balanced approach that considers the intertwined interests of the pregnant person and the fetus. An ethical framework should move beyond this two-patient model (i.e., the pregnant person and the fetus are considered separate patients), which can set maternal and fetal interests against each other, to one that recognizes their interdependence.

In the research setting, the pregnant person and their fetus are acknowledged as an interconnected whole, balancing the unique challenges of pregnancy with the ethical considerations in clinical research. An inclusive framework respects the autonomy and rights of the pregnant person and aims to ensure the health of both parties, fostering a more equitable and compassionate approach to reproductive ethics in clinical trials. Based on our experience, 13 this article analyzes the unique ethical complexities arising in clinical research concerning pregnancy-related medical disorders that predominantly affect fetal well-being.

## The Ethical Framework for Research on Pregnancy-Related Disorders

The ethical framework governing research on pregnancy-related disorders recognizes the importance of safeguarding the health of pregnant persons and their fetus, with the principle of respect for autonomy being paramount. 14 In this context, obtaining the voluntary and informed consent of the pregnant person ensures an alignment of interests between them and their fetus. Choosing to participate in a clinical trial aimed at benefiting the fetus inherently reflects this alignment, reinforcing the ethical commitment to prioritize the health and welfare of both parties.

A principle-based approach combined with a two-patient model 15 has been criticized for setting up a potential conflict between the pregnant person's autonomy and fetal beneficence. 16 However, a principle-based approach does not necessarily assume such conflict, either logically or practically. Rather, a principle-based approach can support the interdependence between the pregnant person's health and autonomy, and potential benefits to the fetus. 17 In addition, a principle-based ethical framework grounded in autonomy, beneficence, non-maleficence, and justice ensures that research on pregnancy-related disorders remains ethically rigorous, respects the dignity, rights, and well-being of the pregnant person and addresses the critical health concerns related to pregnancy-related disorders. Furthermore, safeguarding the interests of pregnant persons also promotes public trust in the ethical conduct of clinical research on pregnancy-related disorders.

### The Principle of Autonomy (Respect for Persons)

Foundational concepts of autonomy and decision-making 18 include control and its bearing on the experience of pregnancy, self-determination, and respect for persons. 19 Respect for autonomy means that patients have the right to determine their medical treatments and whether to participate in a clinical trial, given sufficient information and time to consider their options. Autonomous decision-making during pregnancy is complicated by the need to weigh potential benefits and risks for both the pregnant person and their fetus. 20

#### Complexity of Informed Consent

Informed consent involves an ethical obligation to inform the pregnant person about the potential benefits and risks of participating in a clinical trial and to respect their decision. The voluntary and informed consent of the pregnant person is required for research participation.

The necessity of the father's consent is controversial. In the United States, under the Common Rule (45 CFR 46 Subpart B), the consent of the father is required, unless unavailable, for clinical research with the potential of direct benefit for the fetus alone. For research potentially benefiting the pregnant person, the consent of the pregnant person is sufficient. U.S. Food and Drug Administration (FDA) guidance recommends that the subpart B regulations also be applied to FDA-regulated research, although this is not required. 21

### The Principles of Beneficence and Non-Maleficence

In clinical trial design, the principles of beneficence (i.e., optimizing or maximizing benefit) and non-maleficence (i.e., minimizing harm) form the ethical framework for selecting the indication(s), defining the target population, determining inclusion and exclusion criteria, dosing, and the overall design of the clinical trial. The proportionality of potential benefit and risk is the fundamental consideration of an overall research program. 17

### The Principle of Justice

The principle of justice as fairness in research has generally been articulated as those groups who bear the burdens of research should reap the benefits of that research (i.e., the fairness of inclusion). Historically, and especially following the thalidomide and diethylstilbestrol catastrophes, pregnant persons have been excluded from clinical trials. 22 This exclusion has led to the clinical use of drugs to treat pregnant persons absence of adequate data on dosing, safety, and efficacy. 4 Justice as fairness demands the appropriate inclusion of pregnant persons in research to assure a fair distribution of potential benefits and risks of appropriately tested and labeled medical treatments (i.e., the unfairness of exclusion). 19

## Applying the Ethical Principles to the Design of Perinatal Phase 2 and 3 Interventional Trials

The interdependence of the interests of the pregnant person and their fetus is reflected in the design of clinical studies in two rare conditions of pregnancy, HDFN and FNAIT.

### Applying the Principles of Beneficence and Non-Maleficence in the HDFN and FNAIT Programs

Severe HDFN and FNAIT are diseases of the fetus and newborn caused by maternal alloantibodies with potential poor outcomes, including maternal and neonatal morbidity, preterm delivery, fetal or neonatal death, and long-term disabilities. Severe HDFN is caused by transplacental transfer of pathogenic maternal anti-red blood cell (RBC) alloantibodies that bind to fetal RBCs bearing a paternal antigen not expressed by maternal RBCs, causing fetal hemolytic anemia 23 24 and can result in neonatal anemia and hyperbilirubinemia with potential neonatal morbidity and mortality. 24 25 Similarly, FNAIT is a rare, potentially life-threatening disorder caused by the transplacental transfer of pathogenic maternal IgG alloantibodies, which bind to and destroy fetal platelets due to a human platelet antigen (HPA) incompatibility between the pregnant person and their fetus, with resulting thrombocytopenia in the fetus or newborn. 26 27 28 In both cases, the pathogenic alloantibodies and resulting disease present a significant risk to the health and viability of the fetus and newborn compared to the pregnant person, who only bears a risk for mild or moderate complications.

Nipocalimab, a neonatal Fc receptor (FcRn) blocking therapeutic antibody, administered to the pregnant person, is under evaluation for improving outcomes in the fetus and newborn at risk of severe HDFN or FNAIT. Nipocalimab inhibits maternal to fetal transport of IgG antibodies, including pathogenic maternal anti-RBC and antiplatelet alloantibodies, and decreases the levels of maternal IgG, including anti-RBC and antiplatelet alloantibodies, available for transplacental transfer to the fetus. 23

Given the poor outcomes after prior severe HDFN pregnancy(s), the highest risk HDFN cohort of early-onset severe HDFN (EOS-HDFN) was chosen as the initial indication for the study of nipocalimab in pregnancy. 23 29 30 31 32 In the clinical and research settings, the safety of the pregnant person is paramount; however, there is a level of acceptable risk to the pregnant person to achieve the birth of a healthy neonate. 17 The selection of EOS-HDFN for the initial study of nipocalimab for the treatment of fetal disease due to placental transfer of pathogenic maternal IgG alloantibodies was based on the overall risk–benefit ratio for the pregnant person and the fetus. 17

### Phase 2 Trial in Early-Onset Severe Hemolytic Disease of the Fetus and Newborn

Pregnant persons with a fetus at high risk of EOS-HDFN can be identified by their past obstetric history of EOS-HDFN, a fetus bearing incompatible RBC antigen(s) identified through free fetal DNA (ffDNA) testing, and the presence of known maternal anti-RBC alloantibodies to one or more of those RBC antigens. Management of fetal hemolytic anemia requires close monitoring by Doppler measurement of the middle cerebral artery peak systolic velocity (MCA-PSV), timely detection and treatment with intrauterine transfusions (IUTs) or, with neonatal hemolytic anemia, exchange and/or RBC transfusions. Although IUTs are standard of care (SOC), implementation requires specialized expertise and can result in complications, including fetal mortality. 29 33 34 EOS-HDFN results in fetal/neonatal mortality of 12 to 20% from either disease progression or IUT complications. 35 36 With subsequent pregnancies, EOS-HDFN recurs at a high rate with an 88 to 100% risk for IUTs and neonatal transfusion(s). 29 32 33 34 37 38 The acceptable risk–benefit ratio for a study of EOS-HDFN was supported by the availability of a critical threshold of maternal anti-RBC alloantibodies associated with fetal anemia, ffDNA testing in the maternal circulation to confirm an antigen-positive fetus, non-invasive fetal monitoring using MCA_PSV Doppler ultrasound to predict the onset of fetal anemia, and salvage therapy with IUT of compatible red cells considered the SOC should fetal anemia be detected. Finally, non-clinical data supported the potential for a maximum reduction of maternal IgG of 80 to 85% from baseline and insignificant transplacental IgG transfer with maintenance of complete FcRn saturation and blockade, the potential for safety and insignificant drug exposure in the fetus and newborn, and a consistent, predictable exposure–response relationship supporting weekly intravenous dosing regimens to achieve optimal blockade of placental alloantibody transfer and maternal alloantibody reduction. 23 30 31 39

The design of the single-arm, open-label study to evaluate the safety and efficacy of nipocalimab to delay/reduce fetal anemia and IUTs in EOS-HDFN reflects the principles of beneficence and non-maleficence. 13 The high EOS-HDFN recurrence rate with a substantial risk of mortality due to disease or indicated IUTs supports a clinically meaningful endpoint of live birth at 32 weeks of gestational age (GA) without any IUTs. 13 Given the risks of morbidity and mortality from prematurity, in utero disease activity, and IUT treatment, the choice of 32 weeks GA seeks to balance the neonatal and maternal risks of preterm delivery with the risks of continuing the pregnancy. 40

The ethical principle of equipoise, 41 operationalized in this setting as assuring that enrolled pregnant persons continue to receive known effective interventions, is reflected in the protocol, which includes SOC interventions (e.g., MCA-PSV by Doppler/fetal blood sampling by cordocentesis, followed by IUT as rescue). As such, nipocalimab is added to the SOC. 42 Antenatal intravenous immunoglobulin (IVIg), the only other antenatal treatment option, was excluded as it is unapproved and has variable use across referral centers. 43 Further, only centers that do not routinely use IVIg as a standard therapy were selected as research sites.

Other protocol design features to reduce potential safety risks were implemented. 13 Given the expected reduction in maternal IgG following nipocalimab administration, exclusion criteria included a history of immunodeficiency, certain recurrent infections, and active or potentially serious latent viral infections. In addition, participants were required to be up to date on vaccinations. Treatment cessation 2 weeks prior to delivery, with anticipated restoration of IgG levels, followed by IVIg administration several days prior to delivery, increased maternal IgG with some transfer to the fetus prior to birth. Neonatal risk due to decreased passive immunity was mitigated further by protocol-specified thresholds for IVIg supplementation at birth. Finally, breastfeeding was encouraged to provide optimal neonatal immunity. 44 Infants were monitored until 96 weeks of age (∼2 years) for IgG, safety, vaccine responses, and neurodevelopment. 13

### Phase 3 Severe Hemolytic Disease of the Fetus and Newborn Randomized Controlled Study

A phase 3 study in severe HDFN was designed given the results of the phase 2 study in which 54% of participants met the primary efficacy endpoint compared to a historical benchmark of 10%, with 46% of maternal–fetal–neonatal pairs requiring no intrauterine or neonatal transfusions and preliminary evidence for safety including the lack of unusual or unexpected infections in pregnant persons, their fetuses or infants. 13 The larger ( *n* ∼120), blinded, randomized, placebo-controlled phase 3 study is designed to provide pivotal evidence for the efficacy and safety of nipocalimab added to SOC in pregnant persons at risk for severe HDFN. 45 The primary endpoint remains similar to the phase 2 study, given a reported 86% disease recurrence rate in this population. 32 Reflecting the principle of non-maleficence, the protocol retains similar features to mitigate potential risks, although some elements were modified or removed based on phase 2 data (e.g., administration of IVIg following completion of the treatment period was removed). 45 The 2:1 randomization schedule allows two-thirds of pregnant participants to receive nipocalimab, increasing the likelihood of assignment to the active drug rather than placebo. This approach is justified by the (1) predicted poor outcome of pregnancies with a history of severe HDFN, (2) phase 2 data supporting the efficacy of nipocalimab over SOC, and (3) lack of a feasible active comparator such as IVIg. IVIg is an unproven treatment without well-established response rates and has common and potentially severe side effects that could have a negative impact on the health of the pregnant person. 46 As such, the phase 3 study design reflects the ethical principle of equipoise and the requirement to generate socially valuable data to support the efficacy and safety of nipocalimab in severe HDFN. 47

### Fetal and Neonatal Alloimmune Thrombocytopenia

The initial diagnosis of FNAIT is usually suspected after delivery due to the absence of prenatal FNAIT screening. 48 49 50 The clinical presentation of FNAIT can be asymptomatic; mild with self-limiting hematomas, petechiae, or purpura; or severe with life-threatening complications, including intracranial hemorrhage (ICH) and extracranial hemorrhage. 51 52 53 ICH is the most severe bleeding complication of FNAIT and can result in long-term disability or death of the fetus or newborn. 26 51 52

There are two risk groups for FNAIT pregnancies, depending on whether a previous sibling was affected by FNAIT that did not (standard-risk) or did (high-risk) involve ICH. Management of FNAIT in high-risk pregnancies generally involves off-label antenatal IVIg treatment with or without oral steroids, as the risk of severe bleeding events and/or thrombocytopenia is greatest in this patient population. 26 54 The recommended treatment for standard-risk FNAIT pregnancies involves off-label antenatal IVIg treatment, 27 55 56 57 with the exception of Norway, where antenatal IVIg is used only in high-risk pregnancies. 58 59 The Norwegian Management Model (NMM) for a standard-risk (termed “low risk” in Norway) FNAIT pregnancy involves a no-treatment, non-IVIg algorithm using maternal alloantibody levels and fetal ultrasound to monitor for ICH or other bleeding. These clinical data are used for treatment decisions, including timing and method of delivery.

The widespread off-label use of antenatal IVIg in standard-risk FNAIT pregnancies makes a randomized placebo-controlled study in standard-risk FNAIT challenging, as patient and investigator acceptance (i.e., lack of equipoise) of possible assignment to a placebo arm is low. 60 The heterogeneity in IVIg and IVIg/steroid regimens and inability to blind IVIg administration also make a randomized active-controlled study with an IVIg comparator arm extremely challenging. Thus, the proposed FNAIT clinical development program for nipocalimab includes two studies: (1) A multicenter, randomized, placebo-controlled, double-blind study of nipocalimab in pregnant persons with standard-risk FNAIT (limited to Norway and other international sites that have agreed to the NMM protocol) 60 ; and (2) a global multicenter randomized open-label study of nipocalimab or IVIg in pregnant persons with standard-risk FNAIT (identifier: NCT06533098). Following an interim analysis of the placebo-controlled, randomized controlled trial (RCT) supporting the benefit of nipocalimab, pregnant persons with high-risk FNAIT can be enrolled in the open-label trial.

### The Principle of Autonomy (Respect for Persons) in the Context of HDFN and FNAIT

Depending on the assessment of the prospect of direct (i.e., clinical) benefit to the pregnant person and/or fetus, U.S. Department of Health and Human Services (HHS) regulations may require paternal consent for the HDFN and FNAIT protocols. 61 In both HDFN and FNAIT, whether there is a prospect of direct benefit for the pregnant person may depend on the use of a narrow or broader definition of direct benefit. In HDFN, taking a narrow definition of direct benefit (i.e., clinical benefits which arise from the study intervention) could mean the benefits are accrued by the fetus alone. As such, paternal consent, in addition to the consent of the pregnant person, would be required for clinical trial enrollment. 61 Alternatively, avoiding the potential morbidity of an IUT, preterm birth, fetal demise, and/or a cesarean section may be viewed as offering a prospect of direct benefit for the pregnant person as well, and thus paternal consent would not be required. Critics have argued that requiring paternal consent fails to acknowledge the interdependent interests of a pregnant person and their fetus. However, requiring paternal consent could be based on future obligations for the health and well-being of the child. 62 In addition, one study found that the majority of pregnant persons living with or at risk of HIV/AIDS supported a requirement for paternal consent when the potential benefits were to the fetus alone, albeit with concerns. 63 There is a high degree of variability between the laws in local jurisdictions.

## Engagement with the Patient Community

### Initial Engagement and Consent

One ethical issue discussed during the HDFN phase 2 planning was counseling persons of childbearing potential (POCBP) interested in entering the study but who were not yet pregnant. Aware of the fetal effects of their alloimmunization, these POCBP had experienced a previous fetal loss or the risks of an IUT in the early second trimester. To enter the study with the goal of a healthy neonate, the POCBP need to decide to become pregnant again and therefore potentially put another individual's life at risk. Given that the efficacy of nipocalimab was unproven, this decision might result in fetal loss even before IUT treatment would be technically possible. In this setting, the trial investigator's counsel of POCBP about the possible benefits and risks of nipocalimab and the phase 2 trial might have been misconstrued as an incentive to become pregnant to enroll in the trial. Ultimately, a decision was made that unless the patient was previously known to the investigator, the patient's primary health care provider should undertake the initial counseling and then refer the patient to a trial investigator if they were considering pregnancy.

## Selected Regulatory Considerations for Inclusion of Pregnant Persons in Research

Marketing approval of a drug requires substantial evidence of effectiveness from at least one adequate and well-controlled study and supporting evidence to provide sufficient safety and efficacy data to assess potential benefits and foreseeable risks. 64 65 The choice of an appropriate control group is essential to optimize the ability of a clinical trial to demonstrate a drug's efficacy. In some circumstances, this choice may be challenging. 66 67

The design of the HDFN and FNAIT protocols balances the views of the different stakeholders while generating the necessary data for regulatory approval. The phase 2 HDFN nipocalimab trial did not include a concurrent control group (e.g., placebo) in favor of a comparison to an external historical control. Pregnant persons with EOS-HDFN were enrolled so that the results would be interpretable, given the likelihood that IUTs may be necessary absent nipocalimab in this more severe population. 13 The phase 3 HDFN randomized trial includes a placebo arm and will enroll a broader population of pregnant persons with severe HDFN. Given fetal monitoring and the use of IUT as needed, SOC is not withheld from the placebo group, as there are no approved medical interventions. 45

As described earlier, the FNAIT program includes two clinical studies, with the placebo-controlled study conducted in Norway (and other international sites that adopt the NMM), given existing data supporting this approach. 58 59 The open-label trial includes a small IVIg reference arm ( *N* = 10) to better contextualize the nipocalimab results ( *N* = 40). Following an interim analysis of the standard risk RCT, high-risk pregnant patients can be included in the open-label nipocalimab trial to avoid any excessive risks through randomization of high-risk FNAIT pregnancies to placebo.

## Conclusion

Complex bioethical issues arise in the evaluation of a novel therapeutic agent in rare pregnancy-related diseases with the balance of potential benefit–risk considered for both pregnant participants and their fetuses/neonates. While these rare diseases require large multicenter, international studies for sufficient enrollment to fulfill regulatory requirements, variability in clinical management and the use of unapproved therapies without well-established evidence of efficacy can present challenges to study designs that meet regulatory requirements for pivotal and confirmatory evidence.

Knowledge of the clinical course and management of severe HDFN and FNAIT, the non-clinical and non-pregnant human clinical evidence, and input from physicians, patients, and health authorities permitted the design of clinical protocols that satisfy the principles of beneficence and non-maleficence in these rare and complex diseases while generating sufficient evidence to support an application for marketing authorization for nipocalimab in HDFN and FNAIT indications.
